# Usefulness of a Multiplex PCR Assay for the Diagnosis of Prosthetic Joint Infections in the Routine Setting

**DOI:** 10.1111/os.13187

**Published:** 2022-01-02

**Authors:** Álvaro Auñón, Ismael Coifman, Antonio Blanco, Joaquín García Cañete, Raúl Parrón‐Cambero, Jaime Esteban

**Affiliations:** ^1^ Department of Orthopedic Surgery, Bone and Joint Infection Unit IIS‐Fundacion Jimenez Diaz Madrid Spain; ^2^ Networking Research Centre on Infectious Diseases (CIBER de Enfermedades Infecciosas) Madrid Spain; ^3^ Internal Medicine‐Emergencies, Bone and Joint Infection Unit IIS‐Fundacion Jimenez Diaz Madrid Spain; ^4^ Clinical Microbiology, Bone and Joint Infection Unit IIS‐Fundacion Jimenez Diaz Madrid Spain

**Keywords:** Diagnosis, PCR, Prosthetic joint infection, Routine

## Abstract

**Objective:**

To evaluate the usefulness of a multiplex polymerase chain reaction (PCR) assay as a complementary tool in the diagnosis of prosthetic joint infections in the routine setting of a clinical microbiology laboratory, with a special focus on patients at high risk of culture‐negative infections and high suspicion of infection.

**Methods:**

The results obtained in the routine care setting with the use of the commercial multiplex PCR (Unyvero i60©, Curetis AG, Holzgerlingen, Germany) were retrospectively reviewed. The test was performed in samples of patients with suspected prosthetic joint infection, which were also processed for conventional diagnostic methods, including sonication of the implant when possible. Patients selected for the test were those with negative cultures after a 24‐h incubation period.

**Results:**

Ninety‐nine PCRs were performed, 57 of which were diagnostic of infection according to 2018 MSIS criteria. Nine patients received antibiotics within the 15 days prior to the diagnostic procedure. Tested samples included synovial fluid (33), sonication fluid (56) and tissue biopsies (10). The PCR test detected microorganisms in 26 samples: including two cases of polymicrobial infection. Eleven patients were diagnosed by using PCR only. The most frequently detected microorganism in PCR was Coagulase‐Negative *Staphylococcus* in 11 samples, followed by *Staphylococcus aureus* in five. One sample was positive for the bacteria universal primer, included in the 2.0 version of the kit. Only one discrepancy was detected between a negative PCR and a positive culture. One sample was also positive for a resistance marker (detection of mecA gene in a case of methicillin‐resistant *S. aureus* infection).

**Conclusion:**

The incorporation of the Unyvero ITI multiple PCR technique in patients selected by clinical experts is a useful tool for the diagnosis of bone and joint infections in a routine care setting. A close clinical‐microbiological collaboration improves the usefulness of this kit for the management of patients with these infections.

## Graphical abstract

A useful tool that could be applied in selected patients to decrease the number of culture‐negative prosthetic joint infections.

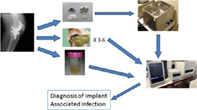

## Introduction

Despite the advances developed during the last decade in the field of the diagnosis of prosthetic joint infection (PJI), the lack of identification of a causative microorganism through traditional culture methods, referred to as culture‐negative infections, remains an issue^1^, ^2^, ^3^, ^4^. Moreover, if other bone and joint infections are considered, the complexity of these procedures increases, especially the interpretation of the results.

One of the most important problems that emerge during patient management is the presence of negative cultures. Even though the incidence of culture‐negative infection decreases when all available methods are applied^5^, there can always be cases where no microorganism is isolated in the different samples and cultures performed.

In an attempt to overcome the problem of culture‐negative PJI, the use of molecular biology appeared as the obvious methodology that could help isolate a micro‐organism, as has been the case with many other fields in microbiology. These techniques allow for the identification of even non‐viable microorganisms by detecting their genetic material. This is of special interest in some patients, such as those under antibiotic therapy before surgery, where conventional techniques have shown their limits in many cases. Many studies have been published about the use of different molecular techniques in the diagnosis of PJI^6^, ^7^, ^8^, ^9^, ^10^, ^11^, ^12^, including several meta‐analyses^13^, ^14^, ^15^. All these studies have shown the usefulness of polymerase chain reaction (PCR) techniques for the diagnosis of orthopedic infections, but no study under routine conditions (usually quite different from experimental ones) has been performed.

During the last years, several studies have shown the characteristics and usefulness of a commercial multiplex PCR kit, Unyvero i60© (Curetis AG, Holzgerlingen, Germany), for the diagnosis of different bone and joint infections^16^, ^17^, ^18^, ^19^, ^20^, ^21^, ^22^, ^23^, ^24^. In a previous study, we evaluated the sensitivity, specificity, positive predictive value, and negative predictive value of this technique^22^. Our results, similar to others', showed excellent performance of the test regarding its specificity, but with low sensibility for the detection of different microorganisms^16^, ^17^, ^18^, ^19^, ^20^, ^21^, ^22^, ^23^, ^24^. Considering the performance of the test, it would be very important to know the actual usefulness of the technique in a routine setting, where the results can be used to guide the management of patients with these infections. In this article we aim to evaluate the usefulness of this methodology in the routine setting of a clinical microbiology laboratory, as a complementary test in our protocol, especially in patients with high risk of culture‐negative infections and a high suspicion index of infection.

## Methods

### General Data

We studied the usefulness of Unyvero i60© after its inclusion for diagnosis of bone and joint infections in the routine setting in October 2015, until June 2018. A retrospective analysis of cases at our institution, a 686‐bed tertiary hospital in Madrid, Spain, was performed. During this period, all samples from 655 patients obtained for diagnosis of PJI were processed using internationally accepted procedures^25^ and a sonication protocol developed in our laboratory^26^. The diagnosis of PJI was performed according to the 2018 MSIS criteria, which are shown in Table 1. Other diagnoses were performed according to well‐known references and guidelines, including erythrocyte sedimentation rate and C reactive protein, aspiration with cell count and cultures^28^, ^29^, ^30^, ^31^. The study was approved by the Ethics in Research Committee from our hospital (CEIC_PIC 45‐2014).

**TABLE 1:** MSIS criteria for prosthetic joint infection

2018 MSIS criteria	
Major criteria (at least one of the following)	Decision
Two positive cultures of the same organism	Infected
Sinus tract with evidence of communication to the joint or visualization of the prosthesis	

Preoperative diagnosis			
Minor criteria		Score	Decision
Serum	Elevated CRP or D‐dimer	2	≥6 infected 4–5 inconclusive ≤3 Not infected
	Elevated ESR	1	
Synovial	Elevated WBC count or LE	3	
	Positive alpha‐defensin	3	
	Elevated synovial PMN(%)	2	
	Elevated synovial CRP	1	

Intraoperative diagnosis		
Inconclusive pre‐op score or dry tap		
	Score	Decision
Preoperative score	—	≥6 infected 2–5 possibly infected 0–1 Not infected
Positive histology	3	
Positive purulence	2	
Single positive culture	2	

**Fig. 1:**
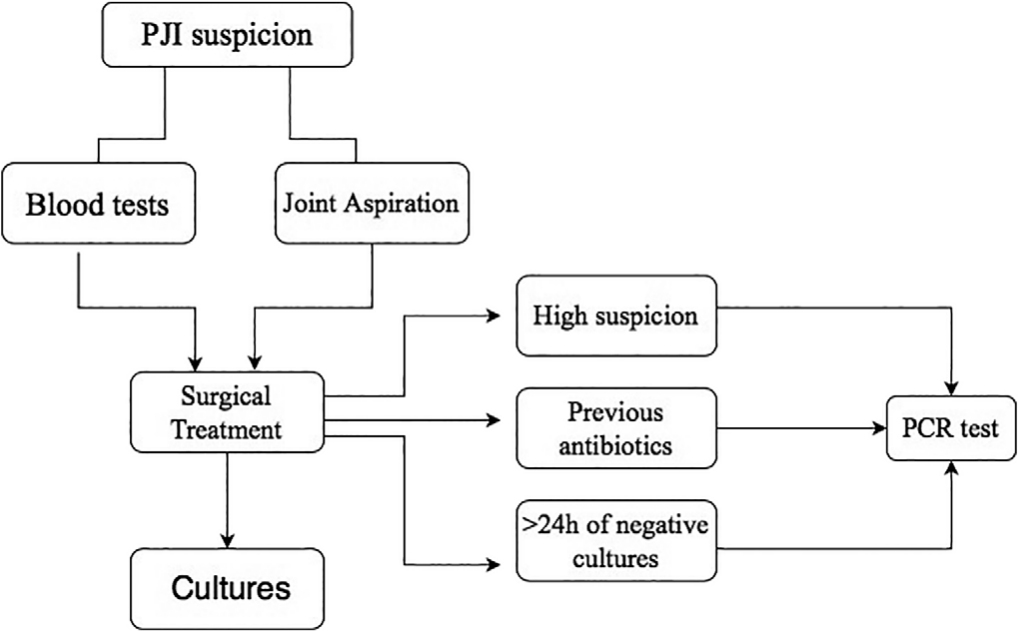
Patient screening flow diagram.

During this period, a PCR test was performed only in those patients with suspicion of infection after consulting between clinicians and microbiologists from the Bone and Joint Infection Unit from our hospital, following a previously established protocol, which includes preoperative blood tests (WBC counts, ESR, CRP), joint aspiration when possible, and microbiological cultures from 5 to 7 samples. After evaluation by a clinician, the use of PCR was discussed with the Microbiology Department and only samples which followed the following criteria were tested: (i) negative result of conventional studies after 24 h; (ii) in patients with clinical findings and 2018 MSIS criteria^27^; compatible with PJI; and/or (iii) having received antibiotic treatment 15 days prior to the sample collection. The institution protocol is shown in Table 2 and Fig. 1.

**TABLE 2:** Institutional protocol for microbiological diagnosis of PJI

Preoperative test	Serum test	ESR, CRP, WBC counts
	Joint aspiration (if possible)	WBC, LE, CRP
Perioperative test	Microbiological cultures: 5–7 cultures	Sonication, synovial fluid, periprosthetic tissue samples
	After 24 h with negative cultures	PCR if compatible with PJI and/or having received antibiotic treatment 15 days prior to the sample collection

CPR, C‐ Reactive Protein; ESR, erythrocyte sedimentation rate test; LE, leukocyte esterase; PCR, polymerase chain reaction; WBC, white blood cell.

### Microbiological Study

The standard microbiological protocol includes the culture of different samples (>3 if possible), including sonication of the implant when available, according to a previously published protocol^26^, and using commonly accepted criteria for interpretation of the results. Briefly, all samples were inoculated on the following media: Tryptic soy‐5% sheep blood agar, Chocolate agar, McConkey agar and Schaedler‐5% sheep blood agar, all from BioMérieux (Marcy l'Etoile, France). Synovial fluid was also inoculated in blood culture bottles (BioMérieux, Marcy l'Etoile, France). Biopsies were processed by mechanic grinding previous to inoculation. Implants were sonicated according to the methodology described in our laboratory^26^, which includes vortexing, sonication and centrifugation previous to inoculation in culture media. All media were incubated for 7 days, and incubation was prolonged to 14 days if suspicion of infection was confirmed by the clinicians. Microbial identification was performed using MALDI‐ToF methodology (Vitek MS©, BioMérieux, Marcy‐l'Étoile, France). This test is able to identify a considerable number of pathogens related to prosthetic joint infections, as shown in Table 3.

**TABLE 3:** List of pathogens/groups of pathogens detected by Unyvero i60ITI kit

*Staphylococcus aureus*	*Escherichia coli*	Other bacteria (universal PCR)
*Coagulase‐negative Staphylococcus*	*Proteus sp*.	*Candida albicans*
*Streptococcus sp*.	*Enterobacter cloacae complex*	*Candida sp*.
*Streptococcus agalactiae*	*Citrobacter freundii/koseri*	*Candida tropicalis*
*Streptococcus pyogenes*	*Pseudomonas aeruginosa*	*Candida glabrata*
*Streptococcus pneumoniae*	*Acinetobacter baumannii complex*	*Candida krusei*
*Granulicatella adjacens*	*Cutibacterium acnes*	*Klebsiella aerogenes*
*Abiotrophia defectiva*	*Finegoldia magna*	*Klebsiella pneumoniae*
*Enterococcus faecalis*	*Bacteroides fragilis complex*	*Klebsiella oxytoca*
*Enterococcus sp*.	*Corynebacterium sp*.	*Klebsiella variicola*

## Results

### General Results

During the study period, samples from 99 patients with suspected PJI were tested for PCR analysis. Tested samples included synovial fluid (33), sonication fluid (56) and tissue biopsies (10). According to clinical, radiological and laboratory parameters, the suspicion of infection was classified as high in 48 cases, moderate in 40 and low in 11. Nine patients received antibiotics in the 15 days prior to the diagnostic procedure. Fifty‐seven patients, according to 2018 MSIS Criteria^27^, were finally diagnosed with infection (34 with previous high suspicion, 21 with moderate suspicion and two with low suspicion). Twenty‐five PCR tests were positive. The final diagnosis was early acute PJI in 14 cases, hematogenous in one case and delayed PJI in 42 cases. The samples were obtained from knee arthroplasty (51), hip arthroplasty (32), elbow arthroplasty (10) and shoulder arthroplasty (6). Data from the patients are shown in the Table S1.

### Microbiological Results

#### Overall Results

The PCR test detected microorganisms in 25 samples; including two cases of polymicrobial infection (two microorganisms were detected in the same sample).

The most detected microorganism was Coagulase‐Negative *Staphylococcus* (CoNS) in 11 samples, followed by *Staphylococcus aureus* (five)*, Cutibacterium acnes* (two) and *Enterococcus sp, Enterobacter cloacae, Streptococcus pyogenes*, *Streptococcus spp, Finegoldia magna, Klebsiella pneumonie, Pseudomonas aeruginosa* and *Candida spp* (one sample each). One sample was positive for the bacteria universal primer, included in the 2.0 version of the kit. No further test could be performed, since the test consists of a closed cartridge system, and prolonged incubation of the culture (15 days) showed no microbial growth.

#### Clinical Relevance

Interestingly, 11 patients were diagnosed according to 2018 MSIS criteria only by using PCR, assuming a PCR positive test equaled a positive culture. Thus, this methodology helped decrease the number of culture‐negative infections from 32 to 21 cases. Considering that the final number of infected patients was 57, the PCR assay accounted for 19.3% of the diagnoses of the cases in which all other techniques gave negative results.

Only one discordant case was detected: one patient with positive PCR for *P. aeruginosa* and with a positive culture for *C. albicans*. Since the introduction of the 2.0 version of the test in 2017 (which includes a universal bacteria well), one case positive for bacteria (without species identification) was detected. One sample tested positive for *Candida spp*., but it was considered a contamination because of the low count and lack of clinical progression without antifungal therapy.

Another aspect of interest was the detection of resistance markers. One case methicillin‐resistant *S. aureus* was detected by PCR. The results of detection of resistance marker correlated with the phenotypic susceptibility of that case.

## Discussion

The diagnosis of bone and joint infection is a complex issue that has improved in the last years by the incorporation of new techniques, such as sonication^32^, prolonged incubation of inoculated media^33^, inoculation in blood culture bottles^34^_,_ and new techniques for microbial identification, such as MALDI‐ToF technology (Vitek MS©, BioMérieux, Marcy‐l'Étoile, France). However, and despite these advances, there are still a group of infected patients that have negative results for all conventional cultures.

For these patients, the obvious answer is the use of molecular biology technology. These methods changed the microbiological diagnosis decades ago, and their use in PJI also started several years ago^9^, ^10^. Only in recent times, commercial techniques designed specifically for the diagnosis of PJI have become available for most laboratories^16^, ^17^, ^18^, ^19^, ^20^, ^21^, ^22^, ^23^, ^24^, ^35^. However, most of these studies were conducted in an experimental setting, designed for a proper evaluation of the technique (including values such as specificity, sensitivity, positive predictive value, and negative predictive value). For this reason, our aim was to study the usefulness of a commercial technique, previously tested by us under experimental conditions^22^, in the routine of a clinical microbiology laboratory. When this technique was introduced in the laboratory, a strict protocol for its use was designed to avoid its misuse. Our protocol establishes that the PCR is to be performed only after careful evaluation of the patient by the members of the multidisciplinary team that manages bone and joint infections in the hospital, and only for patients with high suspicion of infection and a high probability of negative cultures (patients with previous antibiotic therapy or previous negative cultures). In this setting, the technique is expected to yield its maximum efficiency, according to the analysis performed by Liu *et al*.^14^. In previous studies, sensibility of this technique ranges between 48% and 72% (median 67.5%), but specificity appears to be high in all of them (range 92.1%–100%, median 96.3%)^36^. These values have been taken into account for a proper interpretation of the obtained results in the clinical scenario. Moreover, this method proved to be cost‐effective in a pilot analysis^37^.

Our study was not designed to evaluate the method as we have previously performed this analysis^22^, but to evaluate our experience with the use of this methodology in a clinical routine setting using the previously obtained parameters of sensibility and specificity^22^. According to our previous evaluation and other similar reports, specificity is higher than 90%–95%^10^, ^14^, ^16^, ^17^, ^18^, ^21^, ^22^, ^23^, so in the context of a high suspicion of infection, we interpreted a positive result as a true positive. According to our results, the incorporation of this system to our methodology (which includes sonication, the culture of three‐to‐six samples of periprosthetic tissue biopsies and inoculation of liquid samples in blood culture bottles) increases our diagnostic accuracy in 19.3% of the cases (the percentage of culture‐negative infections which were diagnosed only by PCR), supporting the usefulness of this technique as has been demonstrated by other authors for a homemade PCR test^38^. However, this homemade PCR test has the limitation of the need of a specific and experimented molecular biology laboratory, while the commercial systems are designed to be used in less stringent conditions.

Moreover, the relatively high number of PCR‐negative patients with a culture‐positive infections strengthens our idea that a negative PCR result does not exclude an infection, but because the test has a high specificity, a positive result confirms the existence of this syndrome with high accuracy^10^, ^14^, ^16^, ^17^, ^18^, ^21^, ^22^, ^23^. We, as many other authors, believe that using a positive PCR result to affirm the diagnosis instead of a negative PCR result to rule out the infection may improve the diagnostic efficiency and management of these patients. Interestingly, the PCR‐negative, culture‐positive results appear with many different bacterial species, including *S. aureus*, a very important pathogen in this setting, as other authors have described^10^, ^14^, ^16^, ^17^, ^18^, ^21^, ^22^, ^23^, ^24^. The number of bacteria present in the sample does not seem to explain these results, because as we have reported before^22^, some of the cultures have high bacterial counts. The high number of bacterial species, together with the also high number of resistance mechanisms that lead the system to an extreme detection limit could be a potential reason for this lack of sensitivity. Moreover, the presence of a target named “bacteria (other)” opens the necessity of a properly established network of laboratories in order to allow medium and small ones to send these complex cases to the specialized molecular biology laboratories, which unfortunately cannot exist in all clinical microbiology laboratories.

Because we have tried to show the results of the use of this methodology in routine practice, our study has many limitations. The most important being the heterogeneity of the patients and samples, which represents the “real life” of a clinical microbiology laboratory. We have not tried to perform an evaluation of the diagnostic characteristics of the methodology, which have been studied in many reports, including our own. We have used these data of sensitivity, specificity, positive predictive value and negative predictive value for the interpretation of the results of the test, together with the patients' clinical and analytical data. We think that the high specificity of the technique and the fact that all patients have a suspicion of infection allow us to interpret the results as true positives. Both discordant results could be explained by the presence of residual DNA in the case of *P. mirabilis*, and with the interpretation of *C. albicans* isolate as a contamination (it was detected in a very low amount, the patient was not treated with antifungals and further cultures were negative). Other potential causes of discrepancies (such as treatment with antibiotics or detection of fastidious organisms that can be hidden by the growth of other organisms) have not been detected in our data.

More studies are possibly necessary to confirm this assertion, especially because our study has the main limitation of including many different syndromes to be diagnosed and the lack of negative controls. However, this is a study about the clinical usefulness of the technique, and we do not look for a sensitivity/specificity study, but to a clinical relevance study. However, our conclusion is that the use of the commercial PCR test Unyvero i690 ITI with strict conditions for patient selection is potentially useful to improve the diagnosis and management of patients with bone and joint infections in the routine conditions of a clinical microbiology laboratory.

## Supporting information


**Table S1:** Data from all samples and patients with infection.
