# Supplementary material for: Usefulness of a Multiplex PCR Assay for the Diagnosis of Prosthetic Joint Infections in the Routine Setting
Source: Orthop Surg. 2022 Jan 2;14(2):383–8. doi: 10.1111/os.13187 (PMC8867406; doi:10.1111/os.13187)
Supplement: Supplementary file 1 — Table S1: Data from all samples and patients with infection. [file OS-14-383-s001.docx]

| Appendix Table. Data from all samples and patients with infection. | | | | | | | | |
| --- | --- | --- | --- | --- | --- | --- | --- | --- |
|  | Prosthesis | Suspicion of infection | Previous antibiotic | Organism identified in PCR test | Sample | Agent of Infection | 2018 MSIS Score | Infection |
| 1 | Elbow | Low | No |  | Synovial fluid |  | Not applicable | No |
| 2 | Knee | Low | No |  | Synovial fluid |  | 0 | No |
| 3 | Knee | Low | No |  | Synovial fluid |  | 0 | No |
| 4 | Knee | Low | No |  | Synovial fluid |  | 0 | No |
| 5 | Hip | Low | No |  | Sonicate fluid |  | 0 | No |
| 6 | Knee | Low | No |  | Sonicate fluid |  | 2 | No |
| 7 | Knee | Low | No |  | Sonicate fluid | Negative culture | 6 | Yes |
| 8 | Hip | Low | No |  | Sonicate fluid |  | 4 | No |
| 9 | Knee | Low | No |  | Sonicate fluid | Negative culture | 6 | Yes |
| 10 | Knee | Low | No |  | Synovial fluid |  | 3 | No |
| 11 | Knee | Low | No |  | Sonicate fluid |  | 2 | No |
| 12 | Elbow | Moderate | No |  | Synovial fluid |  | Not applicable | No |
| 13 | Knee | Moderate | No |  | Synovial fluid |  | 2 | No |
| 14 | Knee | Moderate | No |  | Synovial fluid |  | 3 | No |
| 15 | Hip | Moderate | No |  | Synovial fluid |  | 3 | No |
| 16 | Knee | Moderate | No |  | Synovial fluid |  | 1 | No |
| 17 | Knee | Moderate | No |  | Synovial fluid |  | 2 | No |
| 18 | Knee | Moderate | No |  | Synovial fluid |  | 1 | No |
| 19 | Hip | Moderate | No |  | Synovial fluid |  | 3 | No |
| 20 | Knee | Moderate | No |  | Synovial fluid | Negative culture | 6 | Yes |
| 21 | Knee | Moderate | No | *S.aureus* | Synovial fluid | *S aureus* | 7 | Yes |
| 22 | Elbow | Moderate | No |  | Synovial fluid | Negative culture | Not applicable | Yes |
| 23 | Knee | Moderate | No |  | Synovial fluid |  | 5 | No |
| 24 | Hip | Moderate | No | *Candida sp.* | Sonicate fluid |  | 3 | No |
| 25 | Hip | Moderate | No |  | Sonicate fluid |  | 2 | No |
| 26 | Hip | Moderate | No |  | Sonicate fluid |  | 2 | No |
| 27 | Knee | Moderate | No |  | Sonicate fluid |  | 2 | No |
| 28 | Knee | Moderate | No |  | Sonicate fluid |  | 3 | No |
| 29 | Knee | Moderate | No |  | Sonicate fluid |  | 2 | No |
| 30 | Knee | Moderate | No |  | Sonicate fluid | Negative culture | 6 | Yes |
| 31 | Knee | Moderate | No |  | Sonicate fluid | Negative culture | 6 | Yes |
| 32 | Knee | Moderate | Yes | *K.pneumoniae* | Sonicate fluid | *K.pneumoniae* | 6 | Yes |
| 33 | Knee | Moderate | No | *P.aeruginosa* | Sonicate fluid | *P.aeruginosa*  *C.albicans* | 6 | Yes |
| 34 | Knee | Moderate | No | CoNS | Sonicate fluid | *S.capitis* | 6 | Yes |
| 35 | Hip | Moderate | No |  | Sonicate fluid | Negative culture | 6 | Yes |
| 36 | Knee | Moderate | No |  | Sonicate fluid | Negative culture | 6 | Yes |
| 37 | Hip | Moderate | No |  | Sonicate fluid | Negative culture | 6 | Yes |
| 38 | Elbow | Moderate | No |  | Sonicate fluid | Negative culture | - | Yes |
| 39 | Shoulder | Moderate | No |  | Sonicate fluid | Negative culture | Possible | Yes |
| 40 | Knee | Moderate | No |  | Sonicate fluid |  | 2 | No |
| 41 | Elbow | Moderate | No |  | Sonicate fluid | Negative culture | Not applicable | Yes |
| 42 | Shoulder | Moderate | No |  | Sonicate fluid | *C.acnes* | Major criteria | Yes |
| 43 | Hip | Moderate | No |  | Tissue biopsy |  | 3 | No |
| 44 | Hip | Moderate | No |  | Tissue biopsy | Negative culture | 6 | Yes |
| 45 | Knee | Moderate | No |  | Synovial fluid |  | 2 | No |
| 46 | Hip | Moderate | No | CoNS | Sonicate fluid | *S.epidermidis* | Major criteria | Yes |
| 47 | Hip | Moderate | No | *Enterococcus sp.* | Sonicate fluid | *Enterococcus sp.* | 7 | Yes |
| 48 | Knee | Moderate | No |  | Sonicate fluid | *S.aureus* | 6 | Yes |
| 49 | Knee | Moderate | No |  | Sonicate fluid | Negative culture | 6 | Yes |
| 50 | Knee | Moderate | No |  | Synovial fluid | Negative culture | 6 | Yes |
| 51 | Knee | Moderate | No |  | Synovial fluid |  | Major criteria | No |
| 52 | Elbow | High | No |  | Synovial fluid | CoNS | Not applicable | Yes |
| 53 | Knee | High | No |  | Synovial fluid | *C.glabrata* | 6 | Yes |
| 54 | Hip | High | Yes | *S. pyogenes* | Synovial fluid | *S. pyogenes* | 6 | Yes |
| 55 | Hip | High | No | CoNS | Synovial fluid | *S.capitis* | Major criteria | Yes |
| 56 | Hip | High | Yes | CoNS | Synovial fluid | *S.epidermidis* | 6 | Yes |
| 57 | Knee | High | No |  | Synovial fluid | *S.epidermidis* | 6 | Yes |
| 58 | Shoulder | High | No |  | Sonicate fluid | Negative culture | Major criteria | Yes |
| 59 | Shoulder | High | No |  | Sonicate fluid | Negative culture | Probable | Yes |
| 60 | Hip | High | No |  | Sonicate fluid |  | 5 | No |
| 61 | Hip | High | Yes | Bacteria | Sonicate fluid | Unknonwn bacteria | Major criteria | Yes |
| 62 | Elbow | High | No | *F.magna* | Sonicate fluid | *F.magna* | Not applicable | Yes |
| 63 | Hip | High | No | *S.aureus* | Sonicate fluid | *S.aureus* | 6 | Yes |
| 64 | Knee | High | No | *S.aureus* | Sonicate fluid | *S.aureus* | 6 | Yes |
| 65 | Elbow | High | No | CNS+*C.acnes* | Sonicate fluid | CoNS+*C.acnes* | - | Yes |
| 66 | Knee | High | No | *Streptpcoccus sp.* | Sonicate fluid | *S.anginosus* | Major criteria | Yes |
| 67 | Hip | High | No |  | Sonicate fluid | Negative culture | 6 | Yes |
| 68 | Hip | High | No |  | Sonicate fluid | Negative culture | Major criteria | Yes |
| 69 | Knee | High | No |  | Sonicate fluid | *S.hominis* | 6 | Yes |
| 70 | Knee | High | No |  | Sonicate fluid |  | 5 | No |
| 71 | Knee | High | No |  | Sonicate fluid | *P.aeruginosa* | Major criteria | Yes |
| 72 | Hip | High | No |  | Sonicate fluid | *S.anginosus* | 6 | Yes |
| 73 | Shoulder | High | Yes |  | Tissue biopsy |  | Probable | No |
| 74 | Elbow | High | No |  | Tissue biopsy |  | Not applicable | No |
| 75 | Shoulder | High | No | *C.acnes* | Tissue biopsy | *C.acnes* | Major criteria | Yes |
| 76 | Elbow | High | No |  | Tissue biopsy | *E cloacae* | Not applicable | Yes |
| 77 | Hip | High | No |  | Sonicate fluid |  | 5 | No |
| 78 | Knee | High | Yes |  | Synovial fluid |  | 5 | No |
| 79 | Hip | High | No |  | Sonicate fluid |  | 5 | No |
| 80 | Knee | High | No | CoNS | Sonicate fluid | CoNS | Major criteria | Yes |
| 81 | Hip | High | Yes | CoNS | Tissue biopsy | *S.epidermidis* | Major criteria | Yes |
| 82 | Knee | High | No | CoNS | Sonicate fluid | *S.hominis* | Major criteria | Yes |
| 83 | Hip | High | No |  | Synovial fluid | *S.epidermidis*  *P.mirabilis* | Major criteria | Yes |
| 84 | Knee | High | Yes |  | Synovial fluid |  | 5 | No |
| 85 | Hip | High | Yes | CoNS | Sonicate fluid | *S.epidermidis* | Major criteria | Yes |
| 86 | Knee | High | No |  | Synovial fluid | *S.epidermidis* | 5 | Yes |
| 87 | Knee | High | No | *E.faecalis* | Synovial fluid | *E.faecalis* | Major criteria | Yes |
| 88 | Knee | High | No | *S.aureus*+CNS | Tissue biopsy | *S.aureus*+CoNS | Major criteria | Yes |
| 89 | Hip | High | No |  | Sonicate fluid | Negative culture | 6 | Yes |
| 90 | Knee | High | No |  | Sonicate fluid |  | 5 | No |
| 91 | Hip | High | No |  | Synovial fluid |  | 3 | No |
| 92 | Knee | High | No |  | Sonicate fluid | *S.epidermidis* | 6 | Yes |
| 93 | Knee | High | No |  | Sonicate fluid | Negative cultures | 6 | Yes |
| 94 | Hip | High | No | MRSA | Tissue biopsy | MRSA | Major criteria | Yes |
| 95 | Knee | High | No | CoNS | Tissue biopsy | *S.epidermidis* | Major criteria | Yes |
| 96 | Hip | High | No |  | Sonicate fluid |  | 2 | No |
| 97 | Knee | High | No |  | Sonicate fluid |  | 3 | No |
| 98 | Hip | High | No |  | Sonicate fluid |  | 3 | No |
| 99 | Knee | High | No |  | Synovial fluid |  | 5 | No |
